# A Change in the Classical Order of Setting of Porous Metal Augments with Locked Cups in Hip Revision Surgery: Technical Note and Case Report

**DOI:** 10.1155/2022/4062172

**Published:** 2022-06-06

**Authors:** Antonio Murcia-Asensio, Francisco Ferrero-Manzanal, Pablo Sanz-Ruiz, Hermenegildo Cañada-Oya, Raquel Lax-Pérez, Christian Goetze

**Affiliations:** ^1^Hospital General Universitario Reina Sofía, Avda. Intendente Jorge Palacios, 1, 30003 Murcia, Spain; ^2^Hospital General Universitario Santa Lucía, C/Mezquita, s/n, Paraje Los Arcos, 30202, Santa Lucía, Cartagena, Spain; ^3^Hospital General Universitario Gregorio Marañón C/Doctor Esquerdo, 46, 28007 Madrid, Spain; ^4^Complejo Hospitalario de Jaén Av. del Ejército Español, 10, 23007 Jaén, Spain; ^5^Auguste-Victoria-KlinikAm, Ruhr-University, Am Kokturkanal 2, 32545 Bad Oeynhausen, Germany

## Abstract

**Introduction:**

Reconstruction of acetabular bone defects by the combination of trabecular metal augments and porous cups can be complex when extensive bone loss and poor-quality bone exists. The onset of porous cups with an interlocking mechanism may simplify surgical technique due to its superior initial mechanical stability. We endorse the possibility for a change in the classical order of setting of the augments and the cup.

**Methods:**

We present a technical modification and a series of cases of three patients with Paprosky IIB and IIIA acetabular defects operated with a combination of porous metal augments and a porous cup. In all the three patients, the setting of the cup was done first and secured with locked screws, and then the augments were set in place as a wedge and fixed with screws in a standard fashion.

**Results:**

The postoperative X-ray showed good position of implants with restoration of the center of rotation, and the patients had good recovery. Radiological evaluation in the midterm follow-up did not show mobilization of implants. *Discussion*. The use of metal porous augments is widely used for severe acetabular defects, being a versatile system to adapt to the different size defects. Nevertheless, its use may be technically demanding and time consuming. It is not infrequent that the setting of the augments conditions the final position of the cup with a possible interference with initial stability and eventually bone ingrowth of the cup. The interlocking mechanism offers an additional biomechanical stability and thus may allow us to place the cup first in the desired position with a less demanding technique.

**Conclusion:**

With the use of locked-screw porous metal cups, the order of setting of implants may be changed in order to obtain a better restoration of the center of rotation and increased host-bone implant contact with a simplified surgical technique.

## 1. Introduction

In hip revision surgery, to achieve a stable and lasting acetabular reconstruction is of paramount importance, mainly in a scenario where extensive bone defects and poor-quality bone exist. The most employed system for assessing acetabular defects is the Paprosky classification and has demonstrated its reliability [1, 2]. According to this classifying method, type I defects are the less demanding ones, with no distortion of acetabular hemispherical shape and minimal bone loss. In type II, there is an oval-shaped acetabulum and is subdivided in types IIA (the superior rim is preserved) and IIB (there is damage of the superior rim with migration of the implant superolaterally) and IIC (deficiency of the medial wall, medial migration of implant). Type III defects are the most demanding, where by definition, there exists insufficient anterosuperior and posteroinferior column support are subdivided in IIIA (when superolateral migration exists, so called “up-and-out”) and IIIB (superomedial migration, so called “up-and-in”). This type of defect requires advanced reconstructive surgical techniques [1, 3, 4]. At the moment of the description of this classification, it was routinely used as a structural allograft for such defects, but nowadays, a variety of surgical strategies have been developed. In the last years, it has been popularized the use of porous metal modular augments in combination with a porous cup, with good published clinical results at the mid- and long-term follow-up [5–7].

Although the use of custom-made cups is getting acceptance, an extensive approach must be performed, and its manufacture requires additional radiological techniques, which are expensive, and delays some time. Besides, the anatomic condition of the patient may be impaired by intraoperative events or may change when the time elapsed between the radiological image and the surgery is long. On the other hand, porous metal modular augments have the advantage of allowing an intraoperative customization of the acetabular reconstruction based on the actual bone defect.

With regard to the order of setting of the augments, there is a wide consensus that the augments must be set in place prior to the insertion of the porous cup, that is, impacted in the reconstructed acetabulum [5, 7–13].

In the last decade, laboratory tests have demonstrated the superiority of locking screws in terms of initial stability between the metal and bone compared to standard nonlocked cups [14, 15]. Recently, there have appeared the “locked cups” that provide a locking mechanism of the screws similar to that of locking plates used in osteosynthesis.

The emergence of this type of implants can make the difference in situations of great size defects, since the motion between the bone and an implant of more than 150 microns can lead to mature connective tissue ingrowth instead of bone ingrowth [16]. We hypothesize that with this type of cups, the order of setting of the porous metal augments and the cup may be inverted.

### 1.1. Case 1

**Figure 1:**
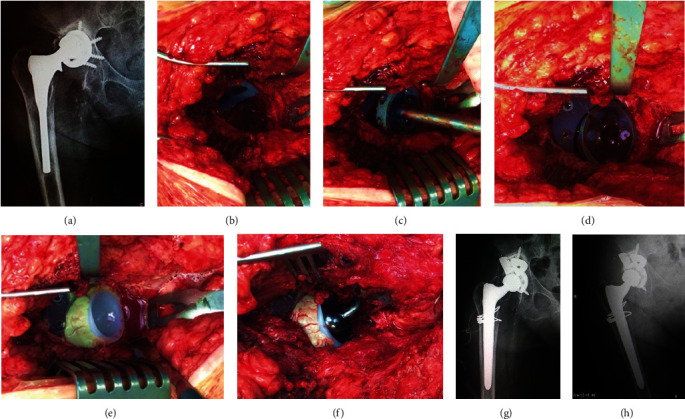
(a) Preoperative X-ray. (b) “Footing” type augment at the cavitary defect. (c) Cup insertion. (d) Cup-augment construct. (e) Polyethylene insertion. (f) Intraoperative image after reduction of the hip. (g) Postoperative X-ray. (h) 5 y follow-up.

The first patient is a 74-year-old lady that presented with hip pain. She had been operated 3 years ago. The radiographic images showed a superior cavitary and superior segmental bone defect that was classified as Paprosky IIIA, with lateral and superior migration of the implant more than 3 cm (Figure 1). Posterolateral approach was performed. The first stage of the surgical procedure consisted of debridement of soft tissue and reaming of the host bone. An augment (Trabecular metal™, Zimmer) was placed at the site of the superior cavitary defect as a foundation or “footing.” Bone allograft was employed to fulfill the existing bone gaps. Careful planification of the acetabular reconstruction was done by using the trial cup and augments. Then, a porous cup (Redapt™, Smith & Nephew) was inserted in the planned position for the adequate correction of the center of rotation. Locked screws at the posterosuperior and posteroinferior location were used to allow for initial stability. The superior segmental defect was then reconstructed by using another “dome-shaped” augment (Trabecular metal™, Zimmer) in an oblong cup position with screws. This augment was cut by using bone shears to fit to the remaining segmental defect. The holes of the cup were protected by plastic plugs to simplify the eventual removal of the construct, except for the adjacent holes to the augment that were intentionally left uncovered to allow for the leakage of the cement to the interface between the cup and the augment at the time of cementation of the polyethylene insert. Partial weight bearing was allowed in the postoperative period. The follow-up at 5 years shows no radiolucent lines and no migration of implants (Figure 1).

### 1.2. Case 2

**Figure 2:**
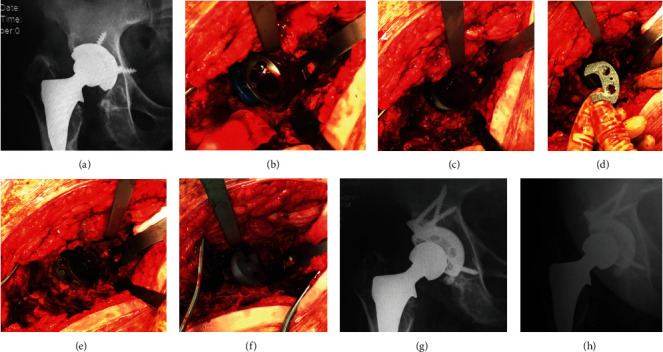
(a) Preoperative X-ray. (b) Simulation of reconstruction with trial implants. (c) Insertion of the cup. (d) “Staple” type implant. (e) Construct after augment implantation. (f) Polyethylene insertion. (g) Postoperative X-ray. (h) 5-year follow-up X-ray.

The second patient is a 72-year-old man that presented a Paprosky IIB defect, with a posterosuperior cavitary/segmental defect (Figure 2). A posterolateral approach was used. After debridement and assessing the actual defect, planification of the reconstruction was done with the trial cup and a trial augment. Reaming of the acetabulum at the anatomic position was performed; then, a porous metal cup (Redapt™) was placed first adding locking screws until adequate stability was obtained. Then, a “staple” type porous augment was secured with 2 screws at the posterosuperior acetabular defect, with the ulterior addition of cement in between in a standard fashion. A polyethylene liner was cemented inside the cup with 4 mm of offset lateralization. The follow-up at 5 years showed good osseointegration of the cup with no migration of implants (Figure 2).

### 1.3. Case 3

**Figure 3:**
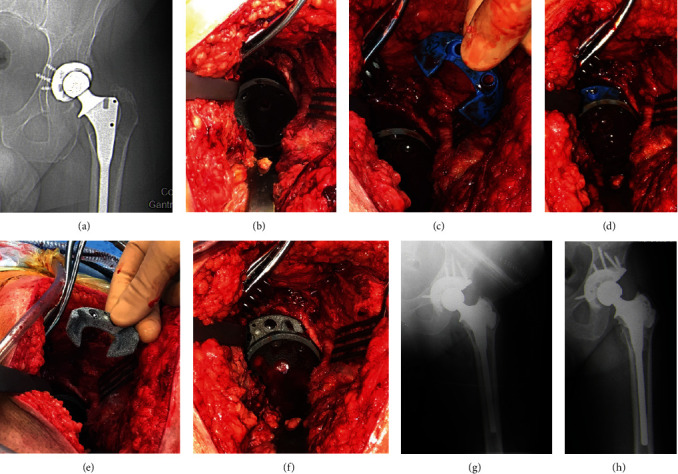
(a) Preoperative X-ray. (b) Intraoperative image after insertion of the cup. (c) and (d) Planification of augment size with trial implants. (e) and (f) Insertion of augment implant (“staple” type). (g) Postoperative X-ray. (h) Two-year follow-up X-ray.

This is a 54-year-old man with painful total hip replacement operated one year before. The radiological images showed a Paprosky IIB defect, with lateral and anterosuperior migration of the implant less than 3 cm (Figure 3). The operation was performed through a posterolateral approach. After debridement and careful reaming of the host bone, planification of the reconstruction of the acetabulum was done with the trial cup and a superior trial augment in an oblong configuration. Then, a porous cup (Redapt™) was inserted in the planned position for anatomic reconstruction of the center of rotation and stabilized with locking screws. Secondly, a porous metal augment (Redapt™, “staple” type) was placed in the upper part of the cup, with interposition of a layer of cement in between at the time of insert cementation. Although the width of the planned trial augment was 18 mm, the final size of the porous augment was smaller. Initial partial weight bearing was allowed. Follow-up at 2 years showed no migration of the implant (Figure 3).

## 2. Discussion

The goals of acetabular revision surgery are to obtain a biomechanical stable implant allowing adequate bone ingrowth into the implant and to restore the center of rotation [16–18]. Although radiological tests can help us to assess the existing defect, the definitive classification of the bone defect must be done after exposition of the acetabular floor and gentle reaming of the acetabulum at the correct position [8]. For this reason, we think that the modularity that offers the combination of a porous cup with porous augments is essential for reconstructing a wide variety of defects.

It has been proposed that the function of augment is more related to its position than to its shape: when placed at the site of the anterosuperior and posteroinferior columns (large intracavitary defects) contribute to the primary stability of the cup. On the other hand, when placed posterosuperiorly for extracavitary defects, the augments act as supplementary fixation [4].

The order of setting of porous metal augments and cups is well established for acetabular defects: the augments are used first to achieve an initial press fit at the time of insertion of the porous cup [3–5, 7–13]. Nevertheless, the exact fixation of the augments may be technically demanding. As the surface finish of both the augment and cup is different, the final position not always fits the trial configuration, and sometimes may occur with some degree of tilting that may condition the ulterior position of the cup, and sometimes with slight modification of the center of rotation, which is considered of great importance [18]. Although in the described case of this paper we did not used intraoperative radiological study, its use may be advisable at the time of fixation of the cup for adequate restoring of the center of rotation.

When placed posterosuperior, a porous augment may also affect the pressfit of the cup when its final position is not accurate. Besides, when inserted first, the augments may also limit the fixation of the porous cup with screws, interfering with the direction of the screws. Regardless, if its structure may allow being perforated with a specific drill, it can be time consuming and may condition the optimal direction and length of the screws to the host bone. Another possible drawback is that impacting the porous cup over the cement layer at the augment site may displace the cement posteriorly in an uncontrolled manner, limiting the contact of the implant with the remaining bone.

By using a porous cup with locked screws, according to the better initial biomechanical stability that offers, the procedure may be simplified. We think that the order of insertion of the porous metal augments may be changed, inserting the augments after the setting of the cup. This does not mean we can relax the exigence of accurate planification pre- and intraoperative and the utilization of bone allograft to restore bone stock as possible.

The locking mechanism of the screws may also avoid the change of the position of the cup that has been demonstrated with nonlocked screw cups [19, 20], allowing more accuracy on the final version and the center of rotation. This statement is not applicable, of course, to cavitary defects in which foundation augments (“footing”) are employed like in Case 1 that must be set first. In a recent article, by using nonlocked cups, the authors propose as well the use of the cup, first with two screws, then turning back out of the screws 2-3 turns and in a second stage the augment is placed, drilled, and removed. The augment is repositioned with a layer of cement in between, and the screws are tightened [6]. This sequence of cup augment application, nevertheless, seems to be complex and time consuming, and we think it can be simplified with locked cups.

The different size between the trial augment and the definitive augment that happened in Case 2 may be attributed to slight modifications of orientation of the cup. If the augment had been placed first, the position of the implant would probably not be the one that we decided initially.

Considering all this, our preferred technique is to carefully debride the soft tissues and assess the bone defect and to prepare the host bone for the implants, reaming at the site of the position of the augment and at the anatomic position of the cup. Then, planification of the reconstruction is done with trial implants. Then, fixation of the cup is done with locked screws in the desired position with restoration of the center of rotation (at least one posterosuperior and one posteroinferior to avoid tilting of the cup). Then, a porous augment (or more, if necessary) is placed and secured with screws. At the time of polyethylene insert cementation, the cement is introduced through the grooves of the augment to create a cement layer, achieving a unitized construct. We nowadays prefer a locked-screw cup when available. The “staple” type configuration of the augment allows for the screw fixation of the porous cup in the area of maximal grip strength without the inconvenience of drilling through its body. Besides, the frontal ports permit the introduction of cement with the use of a syringe at the augment/shell interface.

In case we need “footing” type augment, we use it before the insertion of the cup. In Case 1, as the defects precluded the fitting of conventional size augments, we decided to cut the porous augments to fit the defect. This off-label combination of trabecular metal augments and a Redapt™ porous shell cup was done, because at the time of the surgery, the specific Redapt™ augments were not available yet.

We consider that the adequate position of the porous shell must be mandatory in hip revision surgery and should not be conditioned by an augment, so it must be placed first, allowing a better restoration of the center of rotation (which is of paramount importance for long-term stability) and a better host bone-implant contact and fixation. Comparing with conventional implants, the initial stability of the locked cup would be superior [14, 15].

## 3. Conclusion

The additional stability of the locked-screw porous cups (“locked cups”) may allow us to safely invert the order of setting of the porous modular augments, allowing us to have a better restoration of the center of rotation and increased host-bone implant contact with a less demanding surgical technique. Additional studies are needed to support this assessment.

## Data Availability

The data used to support the findings of this study are included within the article.
